# Correction: Stimulating the motor development of very premature infants: effects of early crawling training on a mini-skateboard

**DOI:** 10.3389/fped.2025.1723022

**Published:** 2025-12-17

**Authors:** Marie-Victorine Dumuids-Vernet, Vincent Forma, Joëlle Provasi, David Ian Anderson, Elodie Hinnekens, Evelyne Soyez, Mathilde Strassel, Léa Guéret, Charlotte Hym, Viviane Huet, Lionel Granjon, Lucie Calamy, Gilles Dassieu, Laurence Boujenah, Camille Dollat, Valérie Biran, Marianne Barbu-Roth

**Affiliations:** 1 Université Paris Cité, CNRS, Integrative Neuroscience and Cognition Center (INCC), Paris, France; 2 CHArt Laboratory (Human and Artificial Cognition), EPHE-PSL, Paris, France; 3 Marian Wright Edelman Institute, San Francisco State University, San Francisco, CA, United States; 4 Service de Néonatologie, Centre Hospitalier Intercommunal, Créteil, France; 5 Service de Néonatologie, Groupe Hospitalier Paris Saint-Joseph, Paris, France; 6 Service de Néonatologie, AP-HP, Maternité Port Royal, Paris, France; 7 Service de Néonatologie, AP-HP, Hôpital Robert Debré, Paris, France

**Keywords:** early intervention, cerebral palsy, crawliskate, locomotion, neonate, newborn

**Error in figure/table**

In the published article, there was an error in Figure 6 as published. The corrected Figure 6 and its caption appear below.

**Figure 6:**
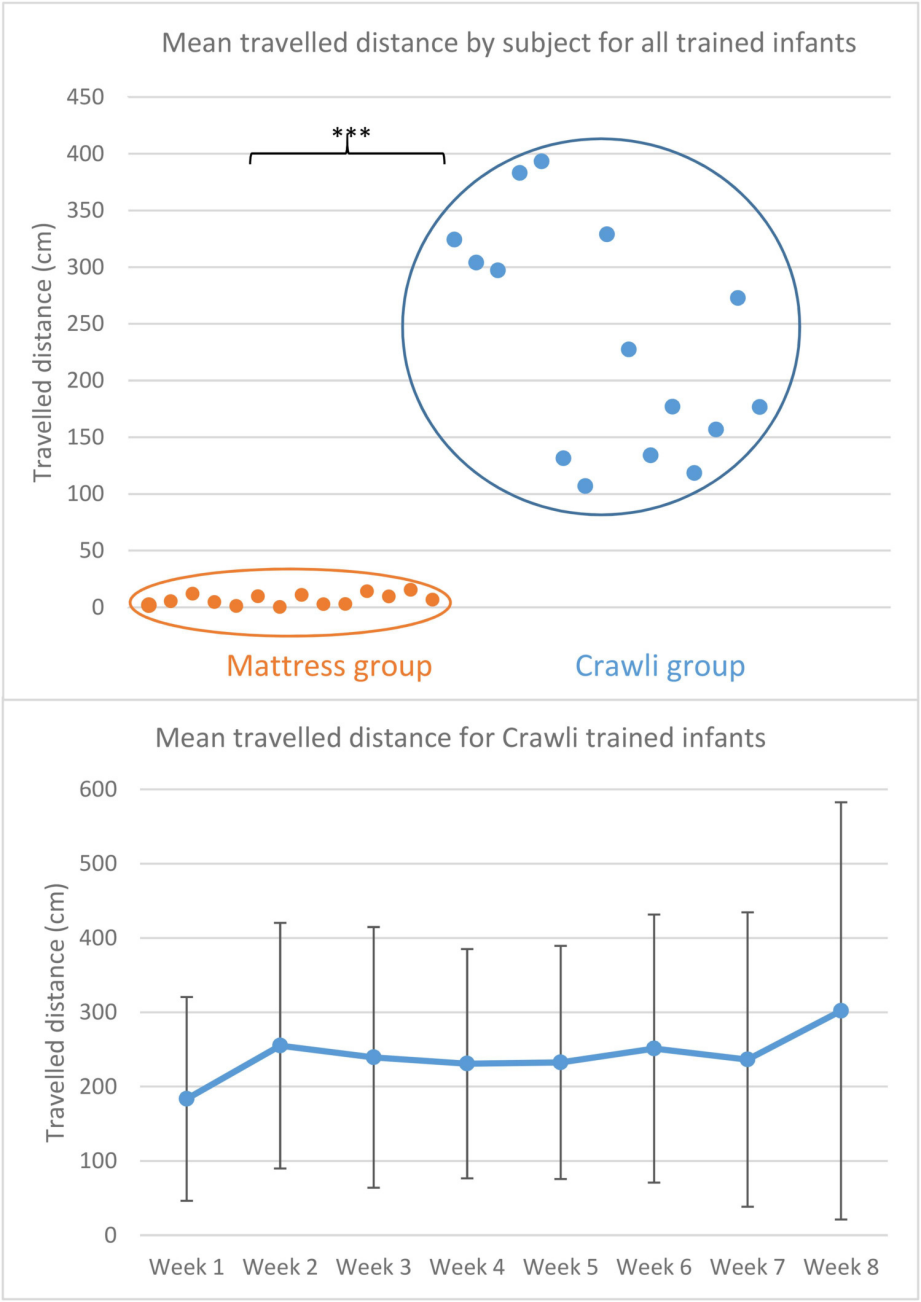
**(A)** Mean distance covered per session by each subject in each group (orange = mattress group; blue = crawli group) during his/her entire training. The distance covered (in cm) is represented on the *y*-axis and the subjects are distributed on the *x*-axis. The *** indicates a *p*-value <0.001. **(B)** Graphical representation of the mean distance (and SD) covered during each of the eight weeks of training for the Crawli group.

**Text correction 1**

In the published article, there was an error.

A correction has been made to **3. Method**, *2.7 Statistical methods*, 2.7.2. Traveled distances during training. This sentence previously stated:

“We compared the mean traveled distances by each infant for all the training sessions between the Crawli and Mattress groups using a student's T-test and reported effect sizes using Cohen's d.”

The corrected sentence appears below:

“We compared the mean traveled distances by each infant for all the training sessions between the Crawli and Mattress groups using a Mann–Whitney U-test and reported effect sizes using rank biserial correlation.”

**Text correction 2**

In the published article, there was an error.

A correction has been made to **3. Results**, *3.3. Training adherence, traveled distances during the sessions and possible harms***,** Paragraph 2. This sentence previously stated:

“All infants trained in the Crawli group were able to move forward on the Crawliskate with a mean traveled distance per session of 138.7 cm (SD = 61.2) and a range from 68.1 to 242.3 cm (see Figure 6A). As expected, in contrast to the Crawli group, infants positioned prone on the mattress were only able to move between 0.12 and 12.7 cm (mean = 6.4 cm, SD = 4.4 cm) (*T* (27) = 8.07, *p* < 0.00001, Cohen's *d* = 3.0 [CI 95%(1.91–4.06)]; Figure 6A).”

The corrected sentence appears below:

“All infants trained in the Crawli group were able to move forward on the Crawliskate with a mean traveled distance per session of 235.4 cm (SD = 58.5) and a range of the mean from 106.8 to 393.3 cm (see Figure 6A). As expected, in contrast to the Crawli group, infants positioned prone on the mattress were only able to move between 0.2 and 15.3 cm (mean = 6.9 cm, SD = 7.6 cm) (*U* = 210, *p* = 0.000005, *r*_rb_ = 1); Figure 6A).”

**Text correction 3**

In the published article, there was an error. A correction has been made to **4. Discussion**, *4.2. Traveled distances during Crawli training*, Paragraph 1. This sentence previously stated:

“It is remarkable that even at this very early age, premature infants could travel long distances with the help of the Crawliskate, up to a maximum of 2.5 meters in only 5 min for some of the infants.”

The corrected sentence appears below:

“It is remarkable that even at this very early age, premature infants could travel long distances with the help of the Crawliskate, up to a maximum of 7 m in only 5 min for some of the infants.”

The original article has been updated.

